# Metabolic Features of a Novel *Trichoderma asperellum* YNQJ1002 with Potent Antagonistic Activity against *Fusarium graminearum*

**DOI:** 10.3390/metabo13111144

**Published:** 2023-11-11

**Authors:** Huimin Ji, Ruohan Yu, Hongyi Liu, Hui Zhang, Xinhua Wang, Jie Chen, Yaqian Li

**Affiliations:** 1 School of Agriculture and Biology, Shanghai Jiao Tong University, Shanghai 200240, China; 2 Key Laboratory of Urban, State Key Laboratory of Microbial Metabolism, Shanghai Jiao Tong University, Shanghai 200240, China

**Keywords:** antagonistic activity, fusarium, metabolomic comparative analysis, *Trichoderma asperellum*, UPLC-MS/MS

## Abstract

*Trichoderma*, a well-known and extensively studied fungal genus, has gained significant attention for its remarkable antagonistic abilities against a wide range of plant pathogens. In this study, a total of 108 *Trichoderma* isolates were screened through in vitro dual antagonistic assays and culture filtrate inhibition against *Fusarium graminearum*. Of these, the YNQJ1002 displayed noteworthy inhibitory activities along with thermal stability. To validate the metabolic differences between YNQJ1002 and GZLX3001 (with strong and weak antagonism, respectively), UPLC-TOF-MS/MS mass spectrometry was employed to analyze and compare the metabolite profiles. We identified 12 significantly up-regulated metabolites in YNQJ1002, which include compounds like Trigoneoside, Torvoside, trans,trans-hepta-2,4,6-trienoic acid, and Chamazulene. These metabolites are known for their antimicrobial properties or signaling roles as components of cell membranes. Enriched KEGG analysis revealed a significant enrichment in sphingolipid metabolism and linoleic acid metabolism, as well as autophagy. The results demonstrated that YNQJ1002’s abundance of antimicrobial substances, resulting from specific metabolic pathways, enhanced its superior antagonistic activity against *F. graminearum*. Finally, YNQJ1002 was identified using the ITS, *tef1-1α*, and *rpb2* regions, with MIST system sequence matching confirming its classification within the species. Overall, we have obtained a novel strain, *T. asperellum* YNQJ1002, which is rich in metabolites and shows potential antagonistic activity against *F. graminearum.* This study has opened promising prospects for the development of innovative *Trichoderma*-derived antifungal compounds, featuring a unique mechanism against pathogens.

## 1. Introduction

*Fusarium graminearum* is a widespread plant pathogen known for causing Fusarium head blight (FHB) in cereal crops [1]. FHB is a devastating fungal disease affecting wheat, oats, barley, and other food crops, leading to seedling blight, ear rot, and crown rot [2]. These infections result in significant economic losses due to a reduction in the quality and output of the crops. For example, in China, *Fusarium graminaria* affects more than 4.5 million hectares of wheat annually on average, accounting for about 20% of the total wheat planting area, and caused yield loss of more than 3.41 million tons per year and an estimated USD 3 billion from 2000 to 2018 [3,4]. Moreover, *F. graminearum* infestation also leads to the production of toxic metabolites that are challenging to remove during grain processing [5]. Consequently, contaminated cereal products pose a serious threat to human health. Therefore, it is imperative to implement effective control and management measures for FHB to protect crop yields and guarantee food safety. 

Numerous management techniques have been employed to control FHB, including the use of resistant varieties, crop rotation, seed treatment, chemical fungicides, and biological control [6,7]. Among them, biological control, due to its environmentally friendly characteristics, has garnered significant attention [8,9]. The use of filamentous fungi *Trichoderma* as biocontrol agents represents an effective alternative for agricultural production systems [10]. The biocontrol mechanism of *Trichoderma* involves a range of strategies, including mycoparasitism, antibiosis, competition for nutrients and space, and induction of plant defense responses, which contribute to its effectiveness in managing plant diseases [11]. Previous studies have shown that *Trichoderma* species, such as *T. gamsii* 6085*, T. harzianum* CCTCC-RW0024, *T. asperellum* GDFS1009, and *T. asperellum* ZJSX5003, have promising in vitro antagonistic activities against *F. graminearum* [12,13,14,15]. Studies have reported that seed treatment with *T. harzianum* can modulate the expression of both ISR- and SAR-type defense genes against *F. graminearum* in maize roots [16]. *Trichoderma* bioactive metabolite treatments on strawberries can increase growth and yield, as well as improve some traits related to fruit quality [17].

It is worth mentioning that *Trichoderma* produces a diverse array of antibiotics and secondary metabolites with antifungal properties [18,19]. These compounds, such as peptaibols, gliotoxin, Harzianic acid, Volatile Organic Compounds, Triterpenes, Trichorzianines, exhibit broad-spectrum activity against various phytopathogens [20]. For instance, the new harziane diterpene harzianol I, a diterpenoid from the fungal symbiont *T. atroviride*, exhibited a potent effect on *Staphylococcus aureus*, *Bacillus subtilis*, and *Micrococcus luteus* [21]. Polyketides diterpenoids from *T. afroharzianum* Fes1712 exhibited selective antifungal activity toward *Botrytis cinerea*, *Fusarium oxysporum*, and *Colletotrichum lagenarium* [22]. Additionally, Trichothecinol A and 8-deoxy-trichothecin produced by *T. longibrachiatum* have shown significant activities toward the soilborne phytopathogen *Colletotrichum lagenarium* [23]. Three peptaibols, trichokonins VI, VII, and VIII, derived from *Trichoderma* koningii, have demonstrated broad-spectrum antimicrobial activity against important plant pathogens, such as *Rhizoctonia solani*, *Fusarium oxysporum*, *Verticillium dahliae*, and *Botrytis cinerea*. Trichokonins are resistant to proteolytic enzymes and exhibit biological activity over a wide pH range, even after autoclaving [24]. Furthermore, Trichobreols A–C, isolated from *T.* cf. *brevicompactum* TPU199, has exhibited antifungal activities against *Candida albicans* and *Cryptococcus neoformans* [25]. By secreting these antimicrobial substances, *Trichoderma* can effectively suppress the growth and development of various competing plant pathogens within the soil environment in the plant rhizosphere. 

In this study, we conducted a large-scale screening process to identify *Trichoderma* strains with good antagonism against *F. graminearum*. Furthermore, we evaluated their cell wall-degrading enzyme (CWDE) activities and antifungal activity using the culture filtrates of *Trichoderma* strains. We performed metabolite profiling for both YNQJ1002 and GZLX3001 (with strong and weak antagonism, respectively) utilizing UPLC-QTOF-MS equipped with an ESI source. KEGG analysis elucidates the significant enrichment of differential metabolites in specific metabolic pathways. Furthermore, YNQJ1002 was conclusively identified as *T. asperellum* using molecular markers, thus shedding light on its potential applications in biocontrol of *F. graminearum* in agriculture.

## 2. Materials and Methods

### 2.1. Fungal Strains and Culture Conditions

All strains and related origins used in this study are listed in Table S1. *Trichoderma* spp. was isolated from farming soils in southern China (Guangdong, Hunan, Guangxi, Tibet, Chongqing, Fujian, Jiangxi, Hainan, and Hubei). The strains of *Trichoderma* spp. and *F. graminearum* were maintained on potato dextrose agar (PDA) at 28 °C for spore harvest. PDA medium was used for confront culture of the pathogens *F. graminearum* and *Trichoderma* spp. [26]. Potato dextrose broth (PDB) medium is the fermentation medium that is typically used for the reproduction of *Trichoderma*. Malt extract agar medium (MEA) was used for *Trichoderma* secondary metabolite production [27].

### 2.2. Dual Confrontation Assays

The in vitro antagonistic properties of the *Trichoderma* strains were investigated using the method described by Szekeres [28]. ImageJ 1.53a was used to calculate the aerial growth of *F. graminearum* in the control and test plates. *Fusarium graminearum* growth inhibition was calculated as a percentage, as described by Saravanakumar [29].

### 2.3. Antifungal Experiment on Strains’ Culture Filtrates

Spores (1 × 10^6^ spores per mL) of *Trichoderma* were inoculated into 100 mL PD liquid media and grown in a thermostatic shaker at 180 rpm at 28 °C for 5 days. The cultures were filtered using a 0.22 µm Minisart^®^ Syringe Filter (Sigma-Aldrich, St. Louis, MO, USA) to obtain cell-free culture filtrate (CF). Subsequently, 100 mL of 5-day culture filtrates and the corresponding PD (controls) was stored at −80 °C for further experimental analysis. Then, 5 mL of CF was added into 10 mL of PDA, mixed well, and the mixture was poured into a culture dish. Plates containing only *F. graminearum* were used as controls. Single agar plugs from the freshly growing mycelium of the specific plant pathogen were inoculated onto the surface of Petri plates (9 cm) containing PDA in the center of the plate.

### 2.4. Assay of Cell Wall-Degrading Enzyme Activity

An enzyme assay was conducted to confirm the presence of cell-free CF of *Trichoderma* spp. Contained cell wall-degrading enzymes (CWDEs) responsible for fungal cell wall degradation, while chitinase, β-1,3-glucanase, and acidic proteinase activities were assayed using the Chitinase, β-1,3-glucanase, and Acidic Proteinase Activity Assay Kit (Solarbio, Beijing, China). An assay with PDB alone served as the control. 

Reducing sugar released in the test reaction mixtures was measured using an ultraviolet/visible light (UV/ViL) spectrophotometer, specifically the UV5300 model from METASH in Shanghai, China. The measurements were taken at wavelengths of 540 nm, 585 nm, and 680 nm for β-1,3-glucanase, chitinase, and acidic proteinase, respectively. Enzymes were assayed in three replicates, and the experiments were repeated twice.

### 2.5. Crude Extraction of Metabolites

The secondary metabolites were produced and extracted using the method reported by Marik et al. [27]. Single agar plugs (5 mm) from the freshly grown mycelium of *F. graminearum* were inoculated onto the surface of Petri plates (15 cm in diameter) containing MEA. The plates were kept at 28 °C for 5 days. Each treatment was carried out in triplicate.

Briefly, the mycelia were harvested using spoons and ground three times with liquid nitrogen. Chloroform (2 × 6 mL per Petri dish) was added to extract peptaibols. After that, chloroform was evaporated to dryness using a vacuum rotary concentrator (Christ, Berlin, Germany) at 45 °C. The crude extracts were dissolved in 1.5 mL of methanol and transferred to new tubes. *Trichoderma* spores and mycelia were pelleted by centrifuging at 12,000 rpm for 2 min using a Heraeus centrifuge (Hanau, Darmstadt, Germany). The supernatant was transferred into new tubes and then evaporated. The remaining dry material was dissolved in 200 µL of methanol and stored at −20 °C.

### 2.6. Analytical Procedures for Peptaibols Using Acquity UPLC-QTOF-MS

UPLC-QTOF-MS was performed using a Waters ACQUITY UPLC system equipped with a Micromass Q-TOF Premier mass spectrometer (Waters MS Technologies, Manchester, UK). Chromatographic separations were performed on a 2.1 × 100 mm (1.7 μm) ACQUITY BEH C18 chromatography column. The column temperature was set at 45 °C, and the gradient eluting program was started with 5% B, changed to 20% B within 2 min, to 100% B within 10 min, then changed to 100% B in another 2 min, to 95% B in 15 min and, at last, held at 95% B for 4 min (Solvent A: aqueous solution of 0.1% formic acid; Solvent B: ACN of 0.1% formic acid). The total flow rate was 0.40 mL/min. The eluate was directed to the mass spectrometer without splitting. Mass analysis was performed using a Q-TOF mass spectrometer equipped with an ESI source operating in the positive and negative ion modes. The desolation and cone gas rates were set at 900 L/h at a temperature of 350 °C and 50 L/h, respectively. The source temperature was set at 115 °C. The collision energy for the MS scan was 6 eV; for the MS/MS scan, the collision energy ramped up from 20 eV to 30 eV. Data were acquired in the centroid mode from the mass-to-charge ratio (*m*/*z*) 50 to 2000 at a scan time of 0.5 s with a lock spray frequency of 15 s, and the acquisition mode used was MSE.

The original LC-MS data were processed with the software Progenesis QI V2.3 (Nonlinear, Dynamics, Newcastle, UK) for baseline filtering, peak identification, integral, retention time correction, peak alignment, and normalization. Main parameters of 5 ppm precursor tolerance, 10 ppm product tolerance, and a 5% product ion threshold were applied. Compound identification was determined based on precise mass-to-charge ratio (*m*/*z*), secondary fragments, and isotopic distribution using the Human Metabolome Database (HMDB), Lipidmaps (V2.3), Metlin, EMDB, PMDB, and self-built databases to conduct qualitative analysis. For the extracted data, ion peaks with missing values within the group all (0 values) > 50% were deleted and the 0 values were replaced with half of the minimum value, and the compounds obtained from characterization were screened according to the compound characterization result scoring (score), which was 36 out of 60, and those below 36 were inaccurate characterization results and were deleted. Finally, the positive and negative ion data were combined into a data matrix, which contains all the information extracted from the raw data that can be used for analyses. The subsequent analyses were based on this matrix. The screening criteria for differential metabolites were VIP > 1.0, FC > 1 or FC < 1, and *p*-value < 0.05. Please refer to the Supplementary Materials for more details on liquid/gas chromatography–mass spectrometry (LC-MS).

### 2.7. DNA Extraction, PCR Amplification, and Sequencing

For the identification of *Trichoderma* spp., the growing mycelium on PDA plates was taken, and DNA was extracted and purified using the FastPure^®^ Plant DNA Isolation Mini Kit (Vazyme, Shanghai, China). The amplicons obtained were confirmed in 1% agarose gel and sequenced in both directions by an external service (Tsinke, Shanghai, China). 

The identification of *Trichoderma* isolates was carried out by amplification and analysis of the regions of the Internal Transcribed Spacer (*ITS*) region of ribosomal DNA (rDNA), Translation elongation factor1-alpha (*tef1-*α), and the second-largest RNA polymerase subunit (*rpb2*) [30]. The amplification was conducted using Taq DNA polymerase, recombinant (Vazyme, China) with the universal primer pairs *ITS*, *tef1*α, and *rpb2* as follows [27]: ITS-4: 5′-TCCTCCGCTTATTGATATGC-3′, ITS-5: 5′-GGAAGTAAAAGTCGTAACAAGG-3′; TEF1-728F: 5′-CATCGAGAAGTTCGAGAAGG-3′,TEF1-LLErev: 5′-AACTTGCAGGCAATGTGG-3′; and RPB2-5f: 5′-GA[T/C]GA[T/C][A/C]G[A/T]GATCA[T/C]TT[T/C]GG-3′,RPB2-7cr: 5′-(CCCAT[A/G]GCTTG[T/C]TT[A/G]CCCAT) 3′.

### 2.8. Molecular Identification and Phylogenetic Analysis

For species identification, a list of *Trichoderma* species and corresponding sequences of reference DNA barcodes can be found in MIST (http://mmit.china-cctc, accessed on 1 September 2022) [30]. The experiments were performed. The sequences used correspond to a concatemer of the ITS (OQ914392), *tef1-*α (OQ988162), and *rpb2* (Q988163) sequences. The phylogenetic tree was constructed using the MEGA-11 program, employing the neighbor-joining method. Bootstrap values were obtained from 1000 replications.

### 2.9. Statistical Analysis

Data were analyzed with ANOVA (two ways) in the statistical package GraphPad Prism version 9.0 for MAC. The enzyme assay and the inhibition rate were response variables with three repetitions. Experiments were validated in duplicate in a completely randomized statistical design. These data were subjected to the Bartlett homogeneity test, and subsequently, a Tukey–Kramer comparison test of means was performed with a probability level of *p* ≤ 0.05.

## 3. Results

### 3.1. Screening for Strains Effective against Fungal Pathogens In Vitro

A total of 108 *Trichoderma* isolates were inoculated on PDA plates and allowed to grow for 5 days. Based on their sporulation and morphological characteristics, the *Trichoderma* strains were categorized into filamentous-type (A) strains (29) and sporogeneses-type (B) strains (79). Subsequently, these 108 *Trichoderma* strains were selected for a dual confrontation test. The results showed that all 108 *Trichoderma* isolates exhibited a different degree of inhibitory effect on *F. graminearum* (Tables S2 and S3, and Figure S1). The inhibition rate of A-type *Trichoderma* ranged from 45.4% to 77.7%. Notably, 15 of the strains displayed inhibitory ratios against *F. graminearum* exceeding 60%. The inhibition rate of B-type *Trichoderma* against *F. graminearum* ranged from 28.4% to 69.3%. Moreover, 13 strains showed an impressive inhibition rate of more than 60%. Both HNSY1005 and SHFX5005 exhibited superior resistance to *F. graminearum* with inhibition rates of 72.86% and 69.8%, respectively.

### 3.2. Effect of Culture Filtrates on F. graminearum

**Figure 1:**
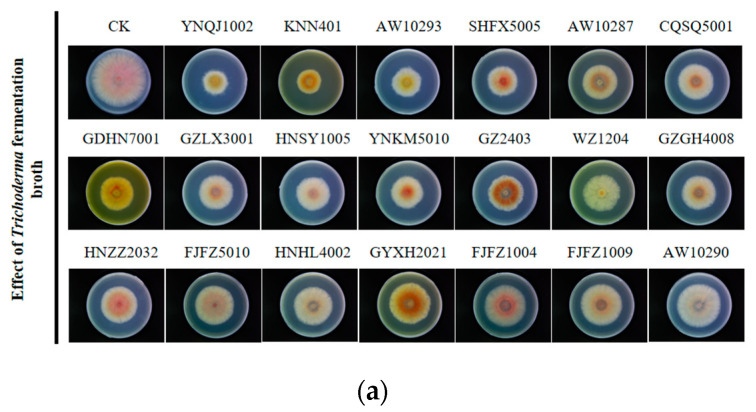

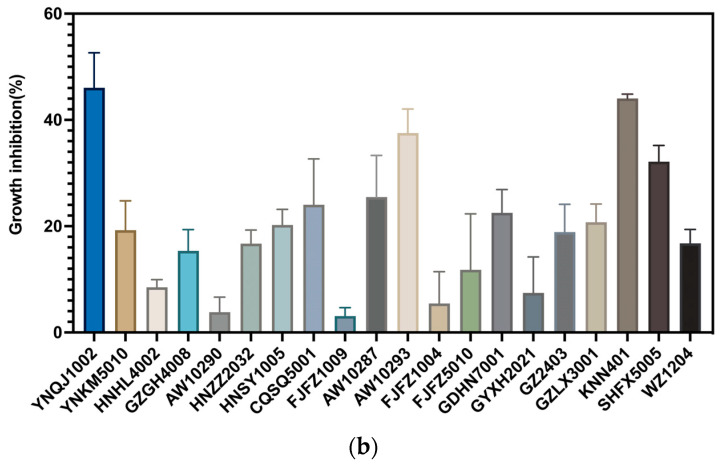
Effect of culture filtrates on *F. graminearum*. (**a**) Inhibition effect of 20 *Trichoderma* isolates’ fermentation liquid on *F. graminearum*; (**b**) inhibition rate of fermentation broth on *F. graminearum*.

Based on previous morphological differences and plate inhibition rate, a total of 20 *Trichoderma* isolates were selected for liquid fermentation, and then, liquid filtrates were blended with PDB to assess their inhibition of *F. graminearum.* As seen in Figure 1, sterile filtrates from different *Trichoderma* isolates showed variations in their ability to resist *F. graminearum*. Among them, the sterile filtrates of YNQJ1002 and KNN401 demonstrated strong inhibitory effects on *F. graminearum*, with inhibition percentages of 46.1% and 44.0%, respectively (Figure 1a,b). These data suggest that the presence of potent antimicrobial substances against *F. graminearum* should be attributed to these two highly inhibitory strains.

### 3.3. Cell Wall-Degrading Enzyme Activities

**Figure 2:**
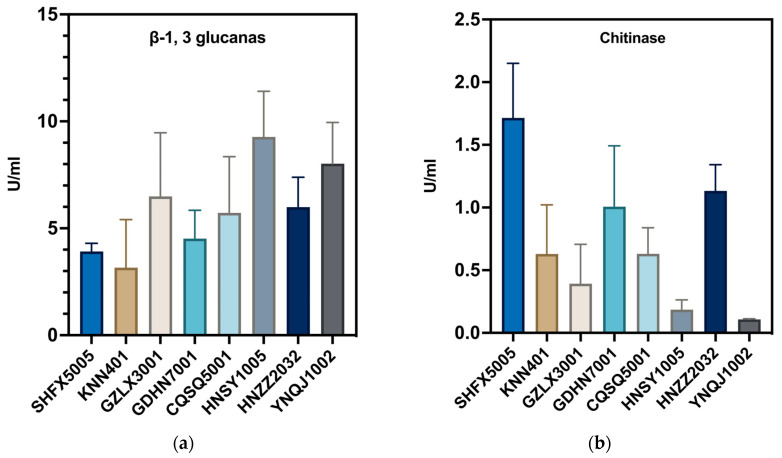

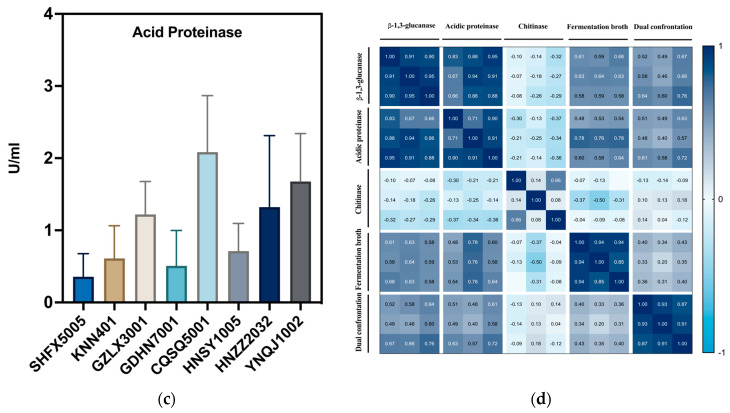
Enzyme assays and statistical analysis. (**a**) Glucanase activity assays for eight *Trichoderma* isolates; (**b**) chitinase activity assays for eight *Trichoderma* isolates; (**c**) protease activity assays for eight *Trichoderma* isolates; (**d**) statistics of correlation coefficient between enzyme activity and inhibition activity.

The secretion of cell wall-degrading enzymes by *Trichoderma* is one of the crucial factors contributing to its antagonistic activity. The activities of three cell wall-degrading enzymes (CWDEs) from eight *Trichoderma* isolates (YNQJ1002, KNN401, GZLX3001, GDHN7001, CQSQ5001, HNSY1005, HNZZ2032, and SHFX5005) were measured to determine which isolates had a good ability to secret the enzymes and attack the cell walls of pathogens. The results indicated that eight *Trichoderma* strains exhibited different abilities in producing CWDEs. Among them, HNSY1005 exhibited the strongest capacity, with a glucanase activity of 9.27 ± 0.23 U/mL, followed by YNQJ1002 with 8.02 ± 0.20 U/mL and GZLX3001 with 6.49 ± 0.18 U/mL. Conversely, KNN401 displayed the lowest ability to produce glucanase, with an enzyme activity of 3.16 ± 0.08 U/mL (Figure 2a).

Regarding chitinase activity, SHFX5005 demonstrated the highest chitinase production capacity, with an extracellular enzyme activity of 1.71 ± 0.43 U/mL, followed by HNZZ2032 (1.13 ± 0.21 U/mL) and GDHN7001 (1.01 ± 0.48 U/mL). Interestingly, YNQJ1002 displayed the weakest ability to produce chitinase, with an enzyme activity of 0.107 ± 0.01 U/mL (Figure 2b). Furthermore, in terms of acid protease production, CQSQ5001 exhibited the highest ability, with an enzyme activity of 2.08 ± 0.78 U/mL, followed by YNQJ1002 with 1.68 ± 0.66 U/mL. In contrast, SHFX5005 showed the lowest capacity to produce acidic protease, with an enzyme activity of 0.36 ± 0.32 U/mL (Figure 2c).

The correlation analysis of several biocontrol-related factors was conducted, and the results indicated a relatively low intergroup correlation between the laboratory-based *Trichoderma*, chitinase activity, glucanase activity, acid protease activity, and their inhibitory rates of fermentation broth and plate confrontation assay. This suggests that under laboratory conditions, each individual enzyme activity could not be consistent with the antagonistic performance, while the secretion of specific metabolites with antagonistic properties is required to achieve a more effective antagonistic action (Figure 2d).

### 3.4. Effect of Crude Extraction of Metabolites on F. graminearum

**Figure 3:**
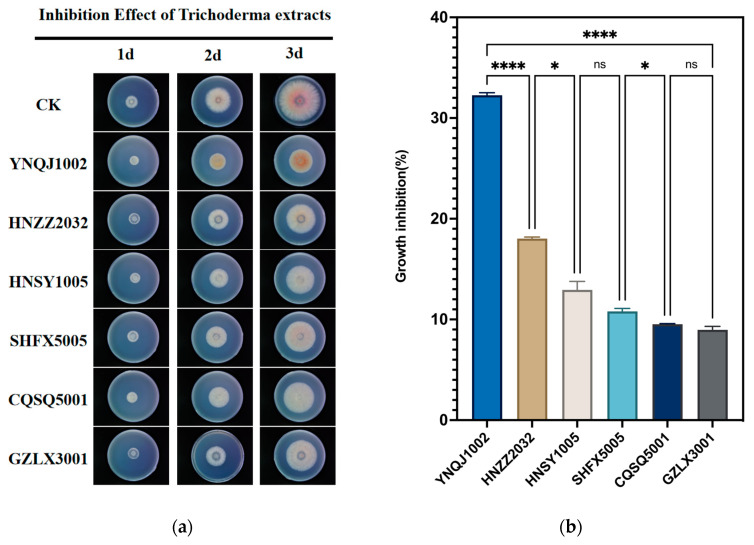
Antagonistic activities of the *Trichoderma* secondary metabolite crude extracts against *F. graminearum*. (**a**) The crude extracts of *Trichoderma* spp. inhibit the mycelial growth form of *F. graminearum*; (**b**) the inhibitory rate of crude extracts on *F. graminearum*. Data are represented as the mean of three replicates ± SD. Stars indicate statistically significant differences (adjusted *p* < 0.01, one-way ANOVA, * *p* < 0.05, **** *p* < 0.0001).

To further confirm the richness and diversity of metabolites secreted by *Trichoderma* and their significant role in antagonizing *Fusarium*, we selected six strains (YNQJ1002, HNZZ2032, SHFX5005, GZLX3001, HNSY1005, CQSQ5001) that exhibited strong antagonistic activity in the dual test for metabolite extraction. The inhibition rate of the crude metabolite extract ranged from 9.5% to 32.3% for each *Trichoderma* strain (Figure 3a). Significantly, *Trichoderma* YNQJ1002 showed the highest inhibition rate, reaching 32.3%, while the lowest inhibition rates were observed for strain GZLX3001 with 9.5%. The analysis of variance (ANOVA) showed significant differences in the inhibition rate among the six strains (Figure 3b).

### 3.5. Analysis of Differential Metabolites between YNQJ1002 and GZLX3001 Using LC–MS/MS

**Figure 4:**
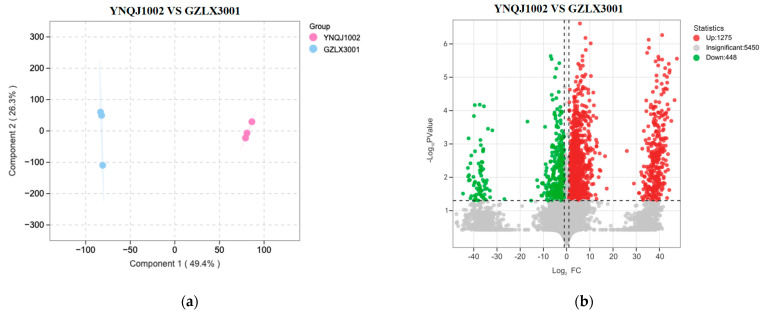

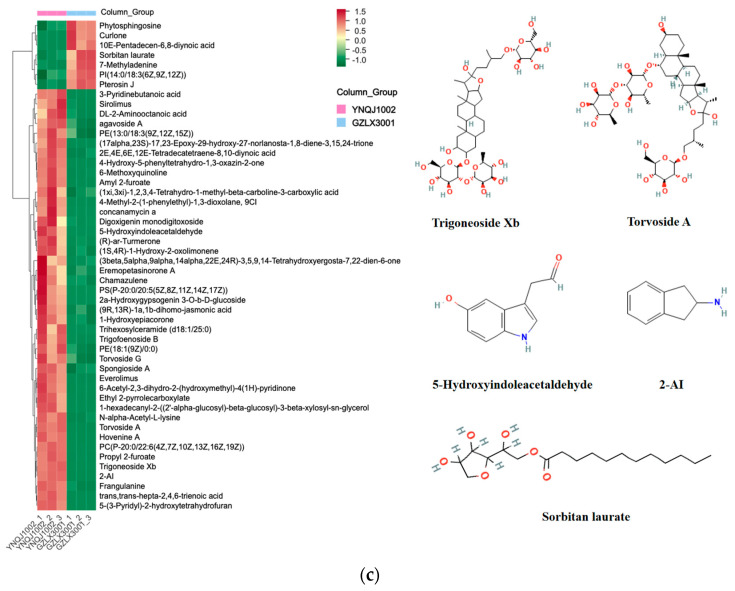
Significantly changed metabolites between YNQJ1002 and GZLX3001 based on LC-MS/MS. (**a**) Principal component analysis (PCA) of YNQJ1002 and GZLX3001; (**b**) volcano plot for differential metabolite analysis in YNQJ1002 and GZLX3001; the red dots are up-regulated metabolites on the right side (fold change > 1) with values greater than 1, the green dots are down-regulated metabolites located on the left side (fold change values < 1); (**c**) heatmap of the top 50 different metabolites between YNQJ1002 and GZLX3001.

In order to demonstrate which antimicrobial substances are responsible for the efficient antagonistic ability of *Trichoderma* against *F. graminearum*, two strains, YNQJ1002 and GZLX3001 with a strong and weak antagonistic ability, were selected for the detection and analysis of differential metabolites using QTOF-UPLC-MS/MS (Figure 4a and Table S5). Principal component analysis (PCA) indicated that the differences in metabolites in individual samples (YNQJ1002 and GZLX3001) are significant.

We identified 1723 compounds that exhibited differences in a comprehensive comparison between YNQJ1002 and GZLX3001. Among these compounds, 1275 were up-regulated and 448 were down-regulated when comparing YNQJ1002 with GZLX3001 (Figure 4b). As shown in Table 1, in the top 50 differential difference compounds, 12 kinds compounds with bioactivity are abundant in YNQJ1002; these substances have been identified as Trigoneoside Xb, 5-Hydroxyindoleacetaldehyde, Everolimus, trans,trans-hepta-2,4,6-trienoic acid, 2-AI, Torvoside A, Trigofoenoside B, 6-Methoxyquinoline, (R)-ar-Turmerone, Frangulanine, Chamazulene, and Torvoside G (Figure 4c). The related information on LC-MS and total ion current and MS/MS for the two strains is shown in Tables S4–S7. Among these compounds, some inhibit fungi and some inhibit bacteria, while others have additional functions such as signaling on the cell membranes or acting as a precursor to the synthesis of valuable secondary metabolites. In contrast, only three antimicrobial compounds in GZLX3001 showed an up-regulated trend.

### 3.6. The KEGG Analysis of Differential Compounds in YNQJ1002 and GZLX3001

**Figure 5:**
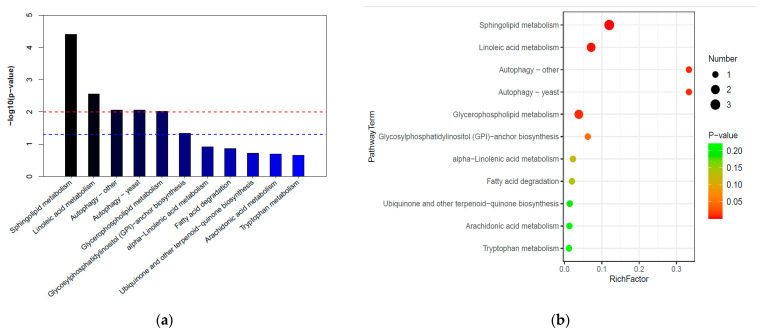
KEGG pathway analysis of the changed metabolites. (**a**) KEGG enrichment bar plot comparing differential metabolites between YNQJ1002 and GZLX3001; (**b**) KEGG enrichment bubble plots comparing differential metabolites between YNQJ1002 and GZLX3001.

A KEGG enrichment analysis was conducted with the differential metabolites generated from the comprehensive comparison between YNQJ1002 and GZLX3001. The calculation of impact and −log10 (*p*) indicated that the different metabolites were involved in a total of 11 metabolic pathways (Figure 5a). According to the rank of impact numerical values, it was found that five metabolic pathways (*p* < 0.01) had the most significant variations, which were related to sphingolipid metabolism and linoleic acid metabolism, autophagy, and glycerophospholipid metabolism. Additionally, the metabolic pathways in which the differential compounds were involved in Ubiquinone and other terpenoid-quinone biosynthesis, Tryptophan metabolism, fatty acid degradation, and Glycosylphosphatidylinositol (GPI)-anchor biosynthesis (Figure 5b).

### 3.7. Molecular Identification and Phylogenetic Analysis

The morphological characterization of YNQJ1002 was carried out after 96 h of growth at 28 °C on PDA. The strain exhibited white, wool-like hyphae and produced green spores from the center, displaying a fast growth rate and an obvious stratification phenomenon. Notably, the inner spore color was dark green, bordering on the outer white zone.

**Figure 6:**
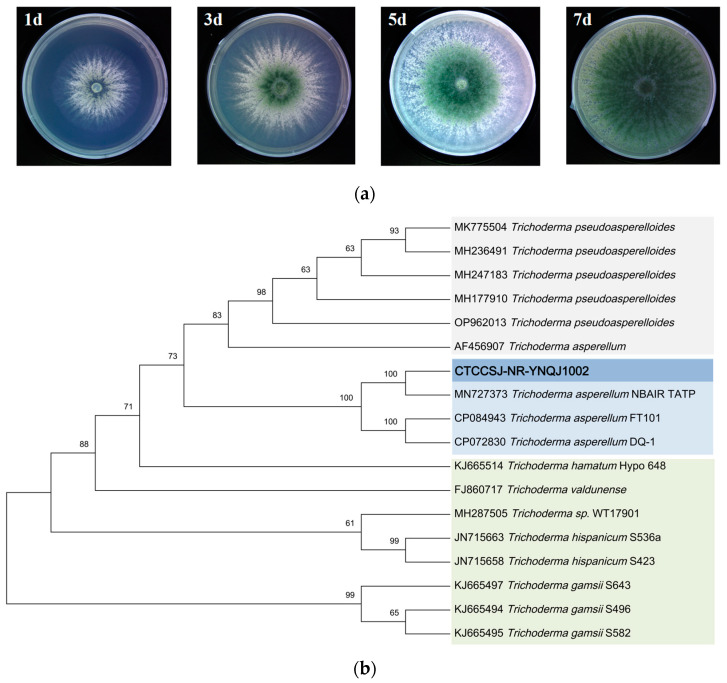
Morphological and phylogenetic analysis of *Trichoderma* YNQJ1002. (**a**) Photograph of colonial morphology on PDA medium; (**b**) phylogenetic tree based on ITS, rpb2, and tef1-1α sequence of *T. asperellum* YNQJ1002 and its closely related species and outgroup retrieved from the literature.

For molecular identification, the ITS (Internal Transcribed Spacer) region, which has been widely used as a criterion for *Trichoderma* species identification, was analyzed using the MIST tool at http://mmit.china-cctc.org, accessed on 1 September 2022 [30]. The ITS sequence identification revealed that YNQJ1002 shared more than 97% sequence homology with 49 species of *Trichoderma*. Subsequently, the tef1-1α sequence was targeted, and YNQJ1002 was found to have 99% homology with *T. asperellum* and *T. kunmingense*. Finally, the *rpb2* sequence analysis further indicated more than 99% homology with *T. asperellum*. The phylogenetic tree further showed a close genetic relationship between YNQJ1002 and *T. asperellum*, as they clustered together in the same branch (Figure 6b). Based on the morphological characteristics and molecular analysis, YNQJ1002 was identified as *T. asperellum*. The GZLX 3001 strain was identified as *T. harzianum* with the same method.

## 4. Discussion

Trichoderma are well-known biological control agents (BCAs) that reduce a range of soilborne pathogen infections and promote plant growth through competition, production of antimicrobial compounds, and mycoparasitism. Previously, research into the screening of *Trichoderma* spp. has been broad, primarily concentrated on dual confrontation for a few strains and physiological as well as biochemical characteristics. Our study was based on large-scale plate confrontation assays, combined with fermentation broth inhibition and cell wall-degrading enzyme assays, as well as liquid chromatography–mass spectrometry (LC-MS) analysis of abundant metabolites from fermentation extracts. Through comprehensive analysis, an antifungal metabolite-rich *T. asperellum* YNQJ1002 was screened and identified as a potential candidate for the biological control of *F. graminearum*.

### 4.1. The Correlation between Cell Wall-Degrading Enzymes and Other Biocontrol Factors

This study began with the assessment of 108 strains of *Trichoderma* using a dual confrontation test. Twenty strains were selected for the antimicrobial assay of their fermentation broth. Among them, eight strains with better antimicrobial characteristics were subjected to enzyme activity assays. The results revealed significant differences in three enzyme activities among the different strains. The stain *T. asperellum* YNQJ1002 exhibited the lowest chitinase activity and better glucanase and protease activities. In contrast, strain *T. harazium* GZLX3001 showed opposite antagonistic characteristics, while its three enzyme activities showed a similar trend to those of YNQJ1002.

The correlation analysis revealed that the correlation among several biocontrol factors was relatively lower. The results indicate that relying solely on plate phenotype, biochemical indicators, or enzyme activity is insufficient for identifying the key factors determining *Trichoderma*’s antifungal activity. These results are consistent with previous reports that showed cell wall-degrading enzymes and secondary metabolites acting synergistically to enhance their biocontrol ability [20]. The interplay between enzyme activity and secondary metabolites can enhance the accessibility of the pathogen’s cell walls to secondary metabolites, thereby increasing the effectiveness of antifungal compounds during the interaction between Trichoderma and pathogens.

### 4.2. Abundant and Specific Metabolites Dominate the Trichoderma’s Antagonistic Action

*Trichoderma* has proven to be a treasure house of interesting secondary metabolites with antifungal properties and medicinal value [18]. To reveal the potential crucial metabolites in *Trichoderma* responsible for *Fusarium* inhibition, we further analyzed the abundant metabolites of the strains through metabolomics and compared the differences between YNQJ1002 and GZLX3001 strains, with strong and weak antifungal abilities, respectively.

The comparative metabolite analysis indicated that a total of 1275 up-regulated compounds were determined through the chloroform/methanol extraction method, aided with LC–MS/MS technology analysis. Among these, 12 significantly up-regulated metabolites were found in YNQJ1002 with diverse biological functions, including the inhibition of certain fungi and bacteria, antioxidant properties, serving as a precursor or signal, and the regulation of plant growth. The results demonstrated a significantly higher abundance of lipid signaling molecules on the cell membranes and antimicrobial substances in YNQJ1002 compared to GZLX3001. This indicates that YNQJ1002 possesses superior bioactive compounds and antagonistic activity against *F. graminearum*. However, these compounds are rarely reported in Trichoderma, but they have been documented in certain plants. These representative compounds that we have selected still require further validation through Nuclear Magnetic Resonance (NMR) spectroscopy, mass spectrometry (MS), and Infrared (IR) spectroscopy.

For instance, Trigoneoside, one of the bioactive compounds isolated from fenugreek seeds, has anticarcinogenic potency through the inhibition of cell proliferation and the inhibition of prostaglandin synthesis [31]. 5-Hydroxyindoleacetaldehyde is a metabolite derived from the metabolism of serotonin, a neurotransmitter and hormone involved in various physiological processes [32]. Trans,trans-hepta-2,4,6-trienoic acid, also known as sorbic acid, is an unsaturated monocarboxylic acid. It is particularly effective in inhibiting the growth of molds and fungi in acidic food products, serving as a preservative to extend the shelf life of these products, and can also be used as a flavoring agent [34]. 2-AI (2-aminoimidazole) and its derivatives are known to have diverse biological activities, including antimicrobial, anti-inflammatory, and antiviral properties [35]. The Torvoside compound was extracted from *S. torvum* leaves, and it was observed that this compound significantly inhibited the growth of all fungi tested [36]. The growth of *A. flavus* and *F. verticillioides* and aflatoxin B1 and fumonisin B1 productions was completely inhibited in vitro and in vivo by *Torvoside K* with increasing concentration [36].

In addition, some compounds play pivotal roles in cellular metabolic processes and possess medical significance. Frangulanine is a natural compound that belongs to a class of compounds known as anthraquinones and has been studied for its potential medicinal properties and its potential anti-inflammatory and antioxidant activities [40]. Chamazulene, an aromatic chemical compound derived from sequiterpenes, is found in nature as a component of many plants and mushrooms. It is well known for its anti-inflammatory and antioxidant properties and has been studied for potential therapeutic uses in traditional and alternative medicine [42]. In comparison, strain GZLX 3001 exhibits a noticeable reduction in the number and variety of up-regulated antimicrobial metabolites, with only three types identified, and has signal transduction and antibacterial activities. By comparing the metabolite profiles of both strains, we found that strain YNQJ1002 is abundant in antifungal metabolites and lipid compounds on the membrane which possibly confer it good ability to inhibit *F. graminearum*.

### 4.3. The KEGG Enrichment of Metabolite Pathways in YNQJ1002

Based on the KEGG database annotations, five metabolism pathways were significantly enriched when comparing YNQJ1002 and GZLX3001. Both sphingolipid metabolism and linoleic acid metabolism are crucial for maintaining cell membrane integrity, regulating cell signaling, and influencing various physiological processes in fungi [46]. They aid in the regulation of membrane potential, the influx and efflux of metabolites, vesicular transport, and in forming membrane signaling domains known as lipid rafts [47,48]. Unsaturated fatty acids (UFAs) and their derivatives, such as linoleic acid and methyl linoleate, have been widely recognized as bioactive fungicides used to control phytopathogenic fungi, including *Fusarium oxysporum* and *Magnaporthe oryzae* [49,50]. Similarly, UFAs inhibit the spore germination, hyphal growth, and pathogenicity of entomopathogenic fungi such as *Beauveria bassiana* and *Conidiobolus coronatus* [51,52]. In addition, we speculated the sphingolipid composition in *Trichoderma* may directly target the sphingolipid metabolism of *F. graminearum*, disrupting their membrane integrity or signaling pathways or triggering apoptotic pathways.

Autophagy is an adaptive response to various stressors, such as nutrient deprivation, oxidative stress, infection, and the buildup of damaged cellular components. It allows cells to survive and adapt to adverse conditions. Autophagy homeostasis plays an essential role in fungal growth and competition, as well as in virulence [53]. The metabolites with antifungal properties in YNQJ1002 could interfere with the autophagic responses of *F. graminearum* directly or indirectly, or trigger signaling pathways that induce the autophagy of the pathogen. Currently, there is relatively little research on the above three metabolic pathways involved in the interaction between *Trichoderma* and pathogens. Understanding these interactions is of paramount importance for the development of biocontrol strategies based on *Trichoderma* in agriculture.

Enriched KEGG analysis revealed a significant divergence in metabolic pathways between YNQJ1002 and GZLX3001. This study not only facilitates the synthesis of antifungal metabolites but also offers exciting opportunities for developing a novel mechanism of action against pathogens. Further efforts will involve optimizing fermentation to isolate and purify one or several metabolites. Alternatively, an analysis of the metabolic gene clusters will be performed, and overexpression vectors will be constructed for heterologous expression in *Saccharomyces cerevisiae* to purify the compounds as potential biocontrol agents in agriculture. Moreover, it would be intriguing to investigate the effects of specific metabolites as signal triggers for the autophagy response in pathogens.

## 5. Conclusions

This work reports a *Trichoderma* strain, *T. asperellum* YNQJ1002, with potentially useful biocontrol activity against Fusarium head blight. *Trichoderma asperellum* YNQJ1002 has a high growth rate, high sporulation capacity, and good glucanease activity. More importantly, *T. asperellum* YNQJ1002 exhibited inhibition rates of 46.1% and 32.3% with its culture filtrates and crude extract, respectively.

Through LC–MS/MS and metabolite profiling analysis, we identified 12 abundant secretions of metabolites; these metabolites belong to fatty acyl glycosides, terpene lactones, sesquiterpenoids, amino acids, peptides, and analogues, among others. KEGG enrichment analysis unveiled significant enrichment in sphingolipid metabolism, linoleic acid metabolism, and glycerophospholipid metabolism, along with autophagy. These pivotal metabolic pathways are involved in the composition and structure of the cell membrane, which significantly regulates the entry and exit of metabolites, influences signaling pathways, and affects the functioning of membrane-bound enzymes.

Based on the present findings, it can be concluded that the enriched metabolites in the cell membrane of *T. asperellum* YNQJ1002 are crucial in the interactions with respective pathogens, potentially altering its growth and development and fitness. Additionally, some of these metabolites may have pharmaceutical value and can be researched and developed into drugs for the treatment of human or animal diseases.

This research holds promise for advancing our understanding of Trichoderma-metabolite-based biocontrol strategies in agriculture and revolutionizing how we combat plant pathogens. However, deeper insights into the mode of action of secreted lipids, peptides, and secondary metabolites involved in plant protection by biocontrol-active *Trichoderma* strains are needed to allow a knowledge-based design of specific biocontrol agents.

## Figures and Tables

**Table 1:** Comparison and functional annotation of differentially antimicrobial metabolites in YNQJ1002 and GZLX3001.

Metabolites	VIP	*p*-Value	log2(FC)	Super Class	Biological Activities
**YNQJ1002**					
Trigoneoside Xb	1.4662	3.96 × 10^6^	44.0422	Fatty acyl glycosides	Antifungal activity [31]
5-Hydroxyindoleacetaldehyde	1.431	1.58 × 10^3^	42.256	Hydroxyindoles	Promote plant growth [32]
Everolimus	1.4621	1.40 × 10^4^	41.554	Unclassified	An mTOR inhibitor [33]
trans, trans-hepta-2, 4, 6-trienoic acid	1.4675	8.87 × 10^6^	38.7295	Fatty acids and conjugates	Antifungal activity [34]
2-AI	1.4683	7.51 × 10^7^	35.3728	Unclassified	Antimicroalgal activity [35]
Torvoside A	1.4699	1.52 × 10^6^	8.294	Terpene lactones	Antifungal and anti-mycotoxigenic potency [36]
Trigofoenoside B	1.4489	5.56 × 10^4^	6.671	Terpene glycosides	Antimicrobial [37]
6-Methoxyquinoline	1.4549	1.23 × 10^4^	6.0625	Unclassified	Antibacterial activity [38]
(R)-ar-Turmerone	1.4233	1.85 × 10^3^	5.4807	Sesquiterpenoids	Induced biochemical defenses [39]
Frangulanine	1.4705	1.74 × 10^5^	5.0688	Amino acids, peptides, and analogues	Antifungal activity [40]
Torvoside G	1.4443	1.11 × 10^3^	3.2429	Triacyclglycerols	Antimicrobial [41]
Chamazulene	1.3990	5.12 × 10^3^	1.9201	Sesquiterpenoids	Antioxidant and radical scavenging activities [42]
**GZLX3001**					
Phytosphingosine	1.4413	5.99 × 10^4^	−1.0791	Amines	Cell proliferation, recognition, adhesion, and signal transduction [43]
Curlone	1.4428	3.07 × 10^4^	−9.2727	Sesquiterpenoids	Antibacterial activity [44]
Sorbitan laurate	1.4364	1.59 × 10^3^	−3.5284	Fatty acid esters	Antibacterial activity [45]

All metabolites’ biological activity is based on the comprehensive metabolite data analysis.

## Data Availability

All data generated during this study are included in the article or Supplementary Materials provided.

## Supplementary Materials

The following supporting information can be downloaded at https://www.mdpi.com/article/10.3390/metabo13111144/s1, Table S1: The original information of 108 Trichoderma strains tested; Table S2: Inhibition effect of 29 A-type *Trichoderma* on *F. graminearum*; Table S3: Inhibition effect of 79 B-type *Trichoderma* on *F. graminearum*; Table S4: Qualitative comprenhensive information of representative metabolites based on the LC-MS/MS; Table S5: The total ion current for strain GZLX3001 and YNJQ1002; Table S6: Tandem Mass Spectra for selected specific compounds in Strian GZLX3001; Table S7: Tandem Mass Spectra for selected specific compounds in Strian YNJQ1002; Figure S1: The phenotype of Trichoderma strains inhibiting the growth of *F. graminearum*.
