# Supplementary material for: Metabolic Features of a Novel Trichoderma asperellum YNQJ1002 with Potent Antagonistic Activity against Fusarium graminearum
Source: Metabolites. 2023 Nov 11;13(11):1144. doi: 10.3390/metabo13111144 (PMC10673152; doi:10.3390/metabo13111144)
Supplement: Supplementary file 1 [file metabolites-13-01144-s001.zip › Supplemetal Figure.pdf]

Figure S1: The phenotype of *Trichoderma* strains inhibiting the growth of *F. graminear um*. (a) The phenotype of 13 sporogenes *Trichoderma* strains with inhibition effect on *F. graminearum*; (b) Inhibition rates of 13 sporogenesis *Trichoderma* strains on the gro wth of *F. graminearu*m; (c) The phenotype of 15 filamentous *Trichoderma* strains inhib iting the growth of *F. graminearum*; (d) Inhibition rate of 15 filamentous *Trichoderma* st rains

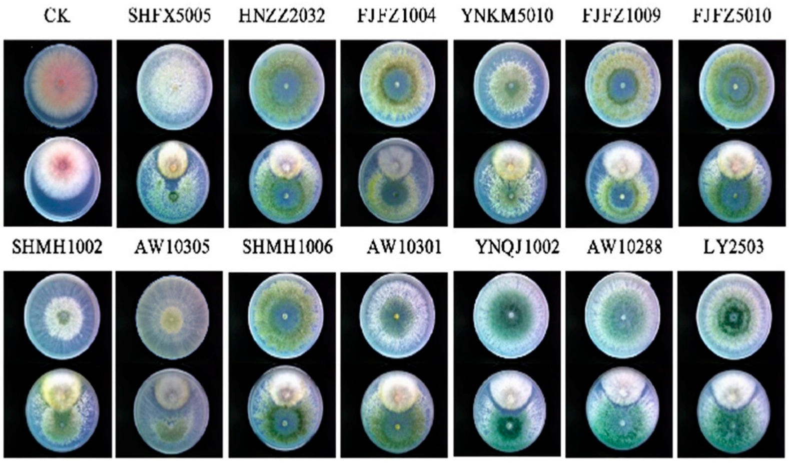

(**a**)

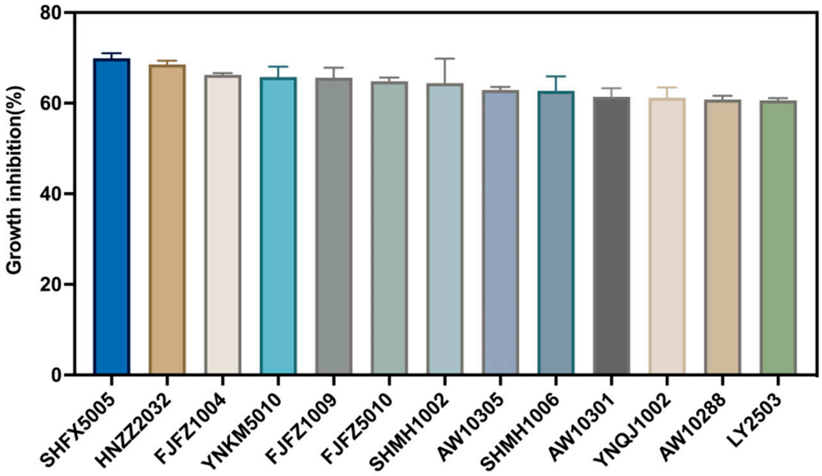

(**b**)

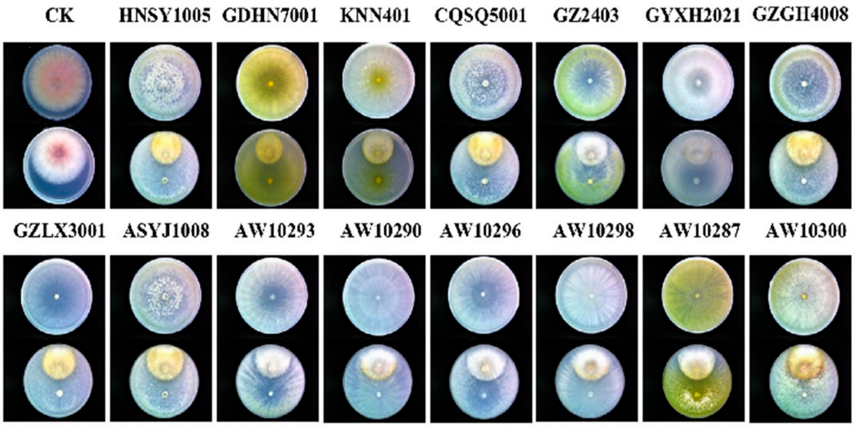
CK
HNSY1005
GDHN7001
KNN401
CQSQ5001
GZ2403
GYXH2021
GZGH4008
GZLX3001
ASYJ1008
AW10293
AW10290
AW10296
AW10298
AW10287
AW10300

(c)

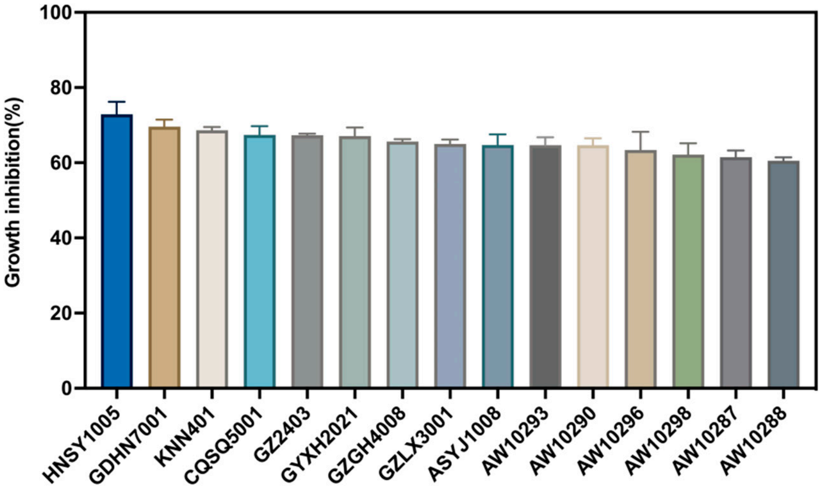
Growth inhibition(%)
100
80
60
40
20
0
HNSY1005
GDHN7001
KNN401
CQSQ5001
GZ2403
GYXH2021
GZGH4008
GZLX3001
ASYJ1008
AW10293
AW10290
AW10296
AW10298
AW10287
AW10288

(d)
